# Minimal Clinically Important Differences With the Outcomes of the App-Based Japanese Allergic Conjunctival Diseases Quality of Life Questionnaire: Cross-Sectional Observational Study

**DOI:** 10.2196/60731

**Published:** 2024-11-26

**Authors:** Ken Nagino, Jaemyoung Sung, Akie Midorikawa-Inomata, Yasutsugu Akasaki, Takeya Adachi, Nobuyuki Ebihara, Ken Fukuda, Atsuki Fukushima, Kenta Fujio, Yuichi Okumura, Atsuko Eguchi, Keiichi Fujimoto, Hurramhon Shokirova, Alan Yee, Yuki Morooka, Tianxiang Huang, Kunihiko Hirosawa, Shintaro Nakao, Hiroyuki Kobayashi, Takenori Inomata

**Affiliations:** 1 Department of Hospital Administration Juntendo University Graduate School of Medicine Tokyo Japan; 2 Department of Ophthalmology Juntendo University Graduate School of Medicine Tokyo Japan; 3 Department of Digital Medicine Juntendo University Graduate School of Medicine Tokyo Japan; 4 Department of Telemedicine and Mobile Health Juntendo University Graduate School of Medicine Tokyo Japan; 5 Department of Dermatology Keio University School of Medicine Tokyo Japan; 6 ENGAGE-Task Force Tokyo Japan; 7 Department of Medical Innovation and Translational Medical Science Graduate School of Medical Science Kyoto Prefectural University of Medicine Kyoto Japan; 8 Department of Ophthalmology Juntendo University Urayasu Hospital Chiba Japan; 9 Department of Ophthalmology and Visual Science Kochi Medical School Kochi University Kochi Japan; 10 Department of Ophthalmology Tsukazaki Hospital Hyogo Japan; 11 Data Science Juntendo University Graduate School of Medicine Tokyo Japan

**Keywords:** allergic conjunctivitis, hay fever, Japanese Allergic Conjunctival Disease Quality of Life Questionnaire, minimal clinically important difference, pollinosis, telemedicine, mobile phone

## Abstract

**Background:**

Assessing changes in quality of life in patients with hay fever–related allergic conjunctivitis requires validated and clinically meaningful metrics. A minimal clinically important difference (MCID) that can be applied to assess Domain II of the Japanese Allergic Conjunctival Disease Quality of Life Questionnaire (JACQLQ) in a smartphone app setting has yet to be determined.

**Objective:**

This cross-sectional observational study aimed to determine MCIDs for the app-based JACQLQ in assessing hay fever–related allergic conjunctivitis.

**Methods:**

This study used data from a crowdsourced, cross-sectional, observational study conducted via the smartphone app “AllerSearch” between February 1, 2018, and May 1, 2020. Participants were recruited through digital media and social networking platforms and voluntarily provided electronic informed consent. Participants completed the JACQLQ, which includes items on daily activity and psychological well-being, as well as a visual analog scale to measure stress levels related to hay fever. Data were collected through the app, ensuring comprehensive user input. MCIDs were determined using both anchor- and distribution-based methods. The face scale of the JACQLQ Domain III and stress level scale for hay fever were used as anchors to estimate the MCID; ranges were derived from these MCID estimates. In the distribution-based method, MCIDs were calculated using half the SD and SE of the JACQLQ Domain II scores. SEs were derived from the intraclass correlation coefficient of an app-based JACQLQ test-retest reliability metric.

**Results:**

A total of 17,597 individuals were identified, of which 15,749 individuals provided electronic consent. After excluding those with incomplete data, 7590 participants with hay fever were included in the study (mean age 35.3, SD 13.9 years; n=4331, 57.1% of women). MCID ranges calculated using the anchor-based method were 1.0-6.9, 1.2-5.6, and 2.1-12.6 for daily activity, psychological well-being, and total JACQLQ Domain II scores, respectively. Using the distribution-based method, the intraclass correlation coefficients were odds ratio (OR) 0.813 (95% CI 0.769-0.849) for daily activity, OR 0.791 (95% CI 0.743-0.832) for psychological well-being, and OR 0.841 (95% CI 0.791-0.864) for total JACQLQ Domain II scores. In addition, the distribution-based method resulted in 2 MCIDs based on half the SD and SE of measurement for daily activity (4.8 and 4.2), psychological well-being (3.4 and 3.1), and total JACQLQ Domain II (7.8 and 6.4) scores. The final suggested MCID ranges for daily activity, psychological well-being, and total JACQLQ Domain II scores were 4.2-6.0, 3.1-4.7, and 6.4-10.5, respectively.

**Conclusions:**

MCID ranges for the JACQLQ estimation could help to standardize the app-based quality of life assessment for patients with hay fever–related allergic conjunctivitis. These MCIDs enhanced the precision of remote symptom monitoring and facilitated timely, data-driven interventions, ultimately improving the overall management and outcomes of allergic conjunctivitis through mobile health platforms.

## Introduction

Hay fever is one of the most common allergic diseases. The global prevalence of hay fever is estimated at 14.4% and is increasing [1,2]. Hay fever is a systemic, multiorgan disease that is associated with diverse comorbidities including allergic conjunctivitis, rhinitis, dermatitis, and asthma [3,4]. Allergic conjunctivitis associated with hay fever is broadly categorized into seasonal and perennial subtypes and comprises a highly multifactorial process that, besides pollen distribution, is mediated by factors such as particulate matter, house dust, dust mites, early childhood pollen exposure, and contact lens use [5-9].

The mainstay of treatment for hay fever with allergic conjunctivitis is post facto symptom management and suppression of exacerbations by antiallergy (antihistamine) or steroid medications [10]. However, owing to the complex interplay of the underlying causal factors and diversity of presentation, optimal treatment and prevention strategies vary from person to person [4]. To improve the individualized treatment of co-occurring hay fever and conjunctivitis, it is essential to conduct a longitudinal evaluation of symptoms and treatment responses derived from patients’ changing symptoms and quality of life (QoL) beyond those presented at the clinic [11].

The holistic evaluation of interventional effects against allergic conjunctivitis typically involves a two-pronged approach that uses patient-reported subjective symptoms and clinician-reported objective findings [12,13]. To best measure subjective symptoms of hay fever that have wide interpatient variance, providers should use validated patient-reported outcomes (PROs) to quantify and standardize the experienced subjective symptoms prior to evaluation [14,15]. PROs are self-reported measurements of the patient’s symptoms, health status, and treatment effectiveness [16,17]. Validated questionnaires that ascertain PROs for subjective symptoms and QoL, enable the objective and consistent quantification and assessment of therapeutic effects [18]. With the rapid advancement in information and communication technology, the collection of electronic PROs (ePROs) through smartphones and electronic devices has increased in clinical settings [19-21]. Research data on the utilization of ePROs in mobile health (mHealth), which uses commonplace mobile smart devices, have become abundantly available [22-26]. Thus, the continuous monitoring of dynamic subjective symptoms and treatment responses of patients through smartphone-based ePROs has potential implications for improving the evaluation of hay fever–induced allergic conjunctivitis, leading to the individualization of treatment regimens.

Accurate assessment of PRO-based disease status and treatment effect evaluation is hindered by the clinical ambiguity of PRO-recorded changes over time [27]. Despite their statistical significance, some differences in PROs may be insufficient to constitute truly “clinically” meaningful and significant changes [28]. Therefore, researchers and providers often apply the concept of minimal clinically important difference (MCID) [27]—a minimal threshold to help determine whether a change in PRO is truly meaningful in clinical practice [29,30]. Thus, a change in the PRO that exceeds a set MCID for an intervention is considered clinically meaningful, regardless of its statistical significance [29]. Numerous reports have indicated that selecting an appropriate MCID requires a prerequisite: assessing the current clinical context and target population to calculate the MCID in a specific setting [27,31]. Therefore, to effectively select an MCID that can determine a meaningful change in PROs, it is essential to evaluate the current clinical context and target population and select an MCID that has been calculated in a similar setting [27].

There are several questionnaires for evaluating hay fever–induced allergic conjunctivitis-related subjective symptoms and QoL. These include the Rhinoconjunctivitis Quality of Life Questionnaire, a disease-specific questionnaire for measuring daily experience related to rhinoconjunctivitis, and the Japanese Allergic Conjunctival Disease QoL Questionnaire (JACQLQ), issued by the Japanese Ocular Allergology Society based on the Japanese Rhinoconjunctivitis Quality of Life Questionnaire (JRQLQ) that was modified for better evaluation of allergic conjunctival disease [15,32,33]. Digitalization of such allergic conjunctivitis-specific questionnaires enables ePRO-based collection of patient symptoms and QoL that allows providers to monitor the disease status remotely. In February 2018, we released an in-house mHealth smartphone app for hay fever research—“AllerSearch”—which collects ePROs from the second domain (Domain II) of the JACQLQ, a 17-item domain that pertains to QoL and is affected by symptoms of hay fever-related allergic conjunctivitis [14,34]. Despite improved accessibility to ePROs for continuous assessment, the absence of a validated MCID constitutes a barrier to the accurate assessment of interval change in the JACQLQ Domain II score. A robust MCID determined in a mHealth environment that targets day-to-day users carries implications for a smartphone app–based platform to nonintrusively evaluate and monitor hay fever status longitudinally in individual patients and the general population [8]. In a clinical study, the MCID was calculated using a paper-based JRQLQ, a predecessor of the JACQLQ [12]. Nevertheless, there are concerns pertaining to the differences in platforms between a traditional paper-based PRO tool and its app-based counterpart, with differences in use conditions, layout, and target users that may affect reporter subjectivity and the final PRO score. Additionally, differences exist between the JRQLQ and JACQLQ in terms of their items and the design of the face scale [12,35]. Accordingly, an MCID that can be applied to assess Domain II of the JACQLQ in a smartphone app setting has not yet been determined.

We previously compared the paper- and app-based JACQLQs and demonstrated the validity and reliability of the app-based JACQLQ [14]. Furthermore, we established the MCIDs for the nasal symptom score, nonnasal symptom score, and total symptom score collected through an app-based questionnaire as indicators for assessing self-reported symptoms of hay fever in a previous study [36]. Upon combining the MCIDs established for the app-based JACQLQ in this study, it will be possible to conduct a detailed assessment of patients with hay fever from the perspective of self-reported symptoms and QoL through a multifaceted approach.

This study aimed to establish the MCIDs and ranges for the electronic version of the JACQLQ Domain II score by analyzing subjective symptoms and QoL data using AllerSearch to improve the current state of smartphone app–based evaluations of hay fever–related allergic conjunctivitis.

## Methods

### Study Design

**Figure 1:**
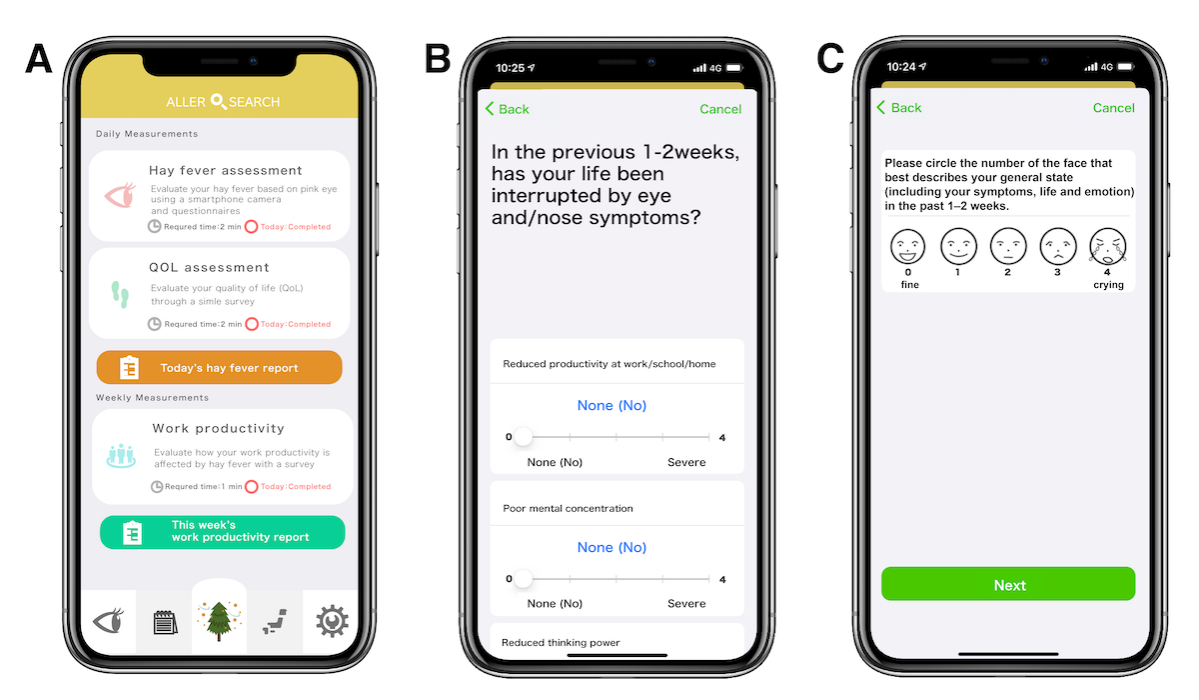
Screenshots of the AllerSearch app. Screenshots of the (A) top screen, (B) JACQLQ Domain II, and (C) face scale score of JACQLQ Domain III. JACQLQ: Japanese Allergic Conjunctival Disease Quality of Life Questionnaire.

This study was performed using data on hay fever from a previous crowdsourced, cross-sectional, observational study that was conducted using the smartphone app “AllerSearch” between February 1, 2018, and May 1, 2020 (Figure 1A) [3,4,37]. In the previous study, participants were recruited through the Apple App Store, and the study was announced via digital media [38] and on social networking platforms (X, formerly known as Twitter [39] and Facebook [40]).

### Ethical Considerations

All procedures involving human participants were performed in accordance with the ethical standards of the institutional or national research committees, in alignment with the 1964 Declaration of Helsinki and its later amendments or comparable ethical standards. The study design was approved by the Independent Ethics Committee of Juntendo University Faculty of Medicine (M17-0061-M02). All participants provided voluntary informed consent electronically [4], with the original consent covering both the primary data collection and any secondary analyses. All study data were anonymized to ensure participant privacy, and no personally identifiable information was included. Participants did not receive any compensation for their involvement in the study.

### Smartphone App “AllerSearch”

“AllerSearch,” a self-developed smartphone app, was released for iOS on ResearchKit in February 2018, and for Android in August 2020, under a consignment contract between Juntendo University Graduate School of Medicine and InnoJin Inc, Tokyo, Japan [3,4,8,41,42]. “AllerSearch” is freely available on the Apple App Store and Google Play.

### Data Source

In this study, data from our previous study using “AllerSearch” were used [4]. The data collection process has been described previously [3,4,8]. Briefly, participants who downloaded “AllerSearch” and provided electronic informed consent shared information on demographics, medical history, lifestyle, and response (nonhay fever, hay fever, and unknown) to the question “Do you have hay fever?” Additionally, they reported their hay fever symptoms and preventive behaviors (Multimedia Appendix 1). The participants then completed questionnaires including the JACQLQ, stress level due to hay fever, nasal symptom score, nonnasal symptom score, and work productivity, which pertained to their daily hay fever symptoms (Multimedia Appendices 2 and 3) [14,32]. Responses to the hay fever symptom questionnaires that were inputted into the app for the first time were used in this study. The stress level was scored on an 11-point visual analog scale ranging from 0 (none) to 10 (most stressful).

The app-based JACQLQ was used in this study to measure QoL in patients with allergic conjunctivitis [14,34,43]. The JACQLQ has validity and reliability based on the results of factor analysis and correlation coefficients [43]. Additionally, the app-based JACQLQ has shown validity and reliability based on internal consistency and the scores of Cronbach α coefficients and correlation coefficients [14]. The JACQLQ comprises three domains: I, II, and III. Domain I comprises 9 items related to ocular and nasal symptoms. Domain II comprises 17 items on daily activity (11 items) and psychological well-being (6 items). Each item in Domains I and II (Figure 1B) is scored on a 5-point Likert scale from none (0 points) to severe (4 points). Domain III consists of a face scale (Figure 1C), which is scored on a 5-point scale to depict emotions from 0 (fine) to 4 (crying) [14,34].

### Inclusion and Exclusion Criteria

Based on the collected data, participants residing in Japan and reporting hay fever were included in this study, whereas individuals without hay fever, with an unknown diagnosis, or with missing questionnaire data related to hay fever symptoms, including the JACQLQ, face scale, and stress level scale, were excluded from this study.

### Anchor-Based Analysis

MCIDs were calculated using both anchor- and distribution-based methods [36]; thus, enabling the use of external indicators as anchors to assign patients to clinically relevant categories [29,44]. It is recommended that the estimation of the MCID in the anchor-based method be based on multiple anchors [45]. Therefore, the face scale from the JACQLQ Domain III and the stress level due to hay fever were used as anchors to determine the MCIDs of the JACQLQ Domain II score.

The face scale of the JACQLQ Domain III comprised a 5-scale anchor to categorize the participants into 5 severity categories [29]. A previous study used a 2-point change as a meaningful difference on an 11-point scale [29,46,47]. Based on this method, an 11-point stress level scale was used as the 5-scale anchor, and participants were assigned to one of the five severity groups [47]. To assess the eligibility of anchors for determining the MCID, the Spearman correlation coefficient and the number of participants in each severity category were calculated [29,47]. The anchor was considered eligible if the correlation coefficient between the anchor and the total score of daily activity, psychological well-being, and total JACQLQ Domain II score was ≥0.3 and the number of participants within a severity category included at least 10 entries [29,45,47]. The mean differences between adjacent severity categories of the anchors provided the MCID estimates. The IQR of the anchor-based MCID estimates was used to calculate the MCID ranges [29,47].

### Distribution-Based Analysis

Two distribution-based methods based on the standard error of measurement (SEM) or half SD were used to provide supporting data in combination with the anchor-based results as follows [36,45]:

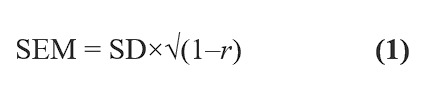



where *r* is the recommended PRO test-retest reliability [27,29]. The intraclass correlation coefficient (ICC) was used as a metric of the test-retest reliability to determine the SEM for calculating MCIDs in this study [27]. ICCs were calculated by comparing baseline and next-day measurements of daily activity, psychological well-being, and total JACQLQ Domain II scores from participants who indicated no change in the face and stress level scale scores [48]. The ICC calculation method was chosen to assess the test-retest reliability of the questionnaire while avoiding symptom changes caused by external factors such as continued pollen exposure, changes in medication use, and variations in pollen dispersal. A PRO score difference that was smaller than the SEM was more likely to represent a measurement error than a significant change [29,45]. Therefore, if the lower limit of the anchor-based MCID ranges were less than the SEM, the SEM was used as the lower limit of the MCID ranges. Half of the SD at baseline was considered equivalent to the MCID [49,50]. The 0.5 SD was calculated in each severity category and compared with the SEM to determine the MCID ranges [51].

### Statistical Analysis

MCID ranges, rather than a single MCID estimate, are recommended for MCID estimation [29]. Therefore, the final MCID ranges were estimated from the MCIDs calculated using the anchor- and distribution-based methods [29]. The final MCID ranges were determined by setting the lower limit as either the 25th percentile, found using the anchor-based method, or the SEM, calculated using the distribution-based method, and the upper limit as the 75th percentile, found using the anchor-based method. To compare the demographics and characteristics of the participants in each severity group, continuous variables were presented as mean and SD, whereas categorical variables were presented as proportions. All statistical analyses were conducted using STATA (version 18.0; StataCorp).

## Results

### Participant Characteristics

**Figure 2:**
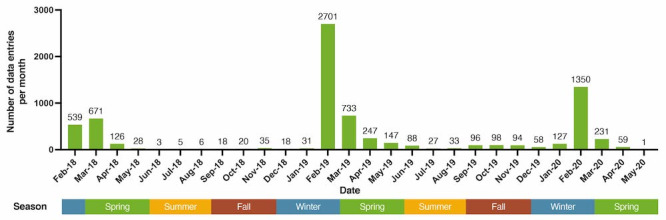
Number of initial data entries during the study period.

A flowchart depicting the screening and enrollment of participants in this study is shown in Multimedia Appendix 4. A total of 17,597 individuals were identified using a unique identifier. Of these, 15,749 individuals provided electronic consent, whereas 1848 individuals did not respond. In total, 11,442 individuals completed the surveys for basic information on characteristics, lifestyle, and hay fever, as well as the surveys for hay fever symptoms and the JACQLQ Domain II. After excluding 113 individuals with outlier data for height, weight, and age, 45 individuals without geographic data, 2243 individuals with nonhay fever or unknown history, and incomplete face scale or stress level surveys, 7590 individuals were included in this study. The demographics and medical history of the 7590 participants with hay fever are summarized in Tables 1 and 2, respectively. The mean age of the participants was 35.3 (SD 13.9) years, and 57.1% (n=4331) of the participants were women. Approximately 82% (6225/7590) of the data related to hay fever were recorded in February or March of each year (Figure 2), which marks the peak months of cedar and cypress pollen distribution in Japan. Based on the scores of both the face scale and stress level scale, participants in the more severe categories were more likely to be younger or female.

**Table 1:** Demographics and medical history by the face scale score category of participants.

				Face scale score 0 (n=667, 8.8%)		Face scale score 1 (n=1864, 24.6%)		Face scale score 2 (n=2755, 36.3%)		Face scale score 3 (n=1849, 24.4%)		Face scale score 4 (n=455, 6%)		Overall (n=7590, 100%)
**Demographics**														
	Age (years), mean (SD)			37.7 (13.8)		37.6 (14.4)		35.4 (13.8)		33.1 (13.2)		30.3 (12.2)		35.3 (13.9)
	Women, n (%)			265 (39.7)		1001 (53.7)		1610 (58.4)		1141 (61.7)		314 (69.0)		4331 (57.1)
	Height (cm), mean (SD)			166.1 (9.0)		164.4 (8.7)		163.5 (8.8)		163.1 (8.8)		162.0 (8.8)		163.8 (8.8)
	Weight (kg), mean (SD)			62.5 (12.3)		60.8 (12.2)		59.8 (12.2)		59.7 (12.4)		58.4 (12.2)		60.2 (12.3)
**Medical history**														
	**Medicated hypertension, n (%)**													
		No	551 (82.6)		1567 (84.1)		2263 (82.1)		1485 (80.3)		359 (78.9)		6225 (82)	
		Medicated	41 (6.2)		122 (6.6)		130 (4.7)		80 (4.3)		13 (2.9)		386 (5.1)	
		Unmedicated	33 (5)		60 (3.2)		91 (3.3)		61 (3.3)		13 (2.9)		258 (3.4)	
		Unknown	42 (6.3)		115 (6.2)		271 (9.8)		223 (12.1)		70 (15.4)		721 (9.5)	
	**Diabetes, n (%)**													
		No	619 (92.8)		1707 (91.6)		2459 (89.3)		1644 (88.9)		390 (85.7)		6819 (89.9)	
		Yes	17 (2.6)		41 (2.2)		62 (2.3)		28 (1.5)		10 (2.2)		158 (2.1)	
		Unknown	31 (4.7)		116 (6.2)		234 (8.5)		177 (9.6)		55 (12.1)		613 (8.1)	
	**Systemic diseases (yes), n (%)**													
		Blood disease	13 (2)		32 (1.7)		39 (1.4)		27 (1.5)		8 (1.8)		119 (1.6)	
		Brain disease	6 (0.9)		15 (0.8)		35 (1.3)		20 (1.1)		4 (0.9)		80 (1.1)	
		Collagen disease	2 (0.3)		8 (0.4)		19 (0.7)		8 (0.4)		4 (0.9)		41 (0.5)	
		Heart disease	18 (2.7)		38 (2)		53 (1.9)		37 (2)		9 (2)		155 (2)	
		Kidney disease	10 (1.5)		33 (1.8)		50 (1.8)		37 (2)		6 (1.3)		136 (1.8)	
		Liver disease	10 (1.5)		34 (1.8)		49 (1.8)		34 (1.8)		5 (1.1)		132 (1.7)	
		Malignant tumor	12 (1.8)		27 (1.5)		35 (1.3)		25 (1.4)		3 (0.7)		102 (1.3)	
		Respiratory disease	39 (5.9)		183 (9.8)		262 (9.5)		203 (11)		66 (14.5)		753 (9.9)	
		N/A^a^	571 (85.6)		1534 (82.3)		2268 (82.3)		1513 (81.8)		371 (81.5)		6257 (82.4)	
	**Atopic dermatitis (yes), n (%)**			91 (13.6)		297 (15.9)		493 (17.9)		367 (19.9)		113 (24.8)		1361 (17.9)
	**Mental illness, n (%)**													
		No	620 (93)		1707 (91.6)		2456 (89.2)		1549 (83.8)		350 (76.9)		6682 (88)	
		Yes	14 (2.1)		69 (3.7)		117 (4.3)		163 (8.8)		63 (13.9)		426 (5.6)	
		Previously had	33 (5)		88 (4.7)		182 (6.6)		137 (7.4)		42 (9.2)		482 (6.4)	
	**History of dry eye diagnosis, n (%)**													
		No	422 (63.3)		1037 (55.6)		1344 (48.8)		873 (47.2)		195 (42.9)		3871 (51)	
		Yes	121 (18.1)		394 (21.1)		678 (24.6)		473 (25.6)		128 (28.1)		1794 (23.6)	
		Unknown	124 (18.6)		433 (23.2)		733 (26.6)		503 (27.2)		132 (29)		1925 (25.4)	

^a^Not applicable.

**Table 2:** Demographics and medical history stratified by the stress level scale category of participants.

					Stress level scale 0-2 (n=2375, 31.3%)			Stress level scale 3-4 (n=1239, 16.3%)			Stress level scale 5-6 (n=1685, 22.2%)			Stress level scale 7-8 (n=1702, 22.4%)			Stress level scale 9-10 (n=589, 7.8%)			Overall (n=7590, 100%)
**Demographics**																				
	Age (years), mean (SD)			39.5 (13.7)			36.0 (14.3)			35.3 (13.5)			30.6 (12.6)			29.9 (12.2)			35.3 (13.9)	
	Women, n (%)			1176 (49.5)			699 (56.4)			941 (55.9)			1104 (64.9)			411 (69.8)			4331 (57.1)	
	Height (cm), mean (SD)			164.9 (8.8)			163.7 (8.7)			163.9 (8.8)			162.7 (8.8)			162.0 (8.7)			163.8 (8.8)	
	Weight (kg), mean (SD)			61.5 (12.2)			60.2 (12.0)			60.2 (12.0)			59.0 (12.3)			58.1 (12.9)			60.2 (12.3)	
**Medical history**																				
	**Medicated hypertension, n (%)**																			
		No	1983 (83.4)			1033 (83.4)			1358 (80.6)			1400 (82.3)			451 (76.6)			6225 (82)		
		Medicated	164 (6.9)			76 (6.1)			86 (5.1)			47 (2.8)			13 (2.2)			386 (5.1)		
		Unmedicated	94 (4)			39 (3.2)			57 (3.4)			53 (3.1)			15 (2.6)			258 (3.4)		
		Unknown	134 (5.6)			91 (7.3)			184 (10.9)			202 (11.9)			110 (18.7)			721 (9.5)		
	**Diabetes, n (%)**																			
		No	2164 (91.1)			1131 (91.3)			1502 (89.1)			1508 (88.6)			514 (87.3)			6819 (89.9)		
		Yes	69 (2.9)			22 (1.8)			33 (2)			28 (1.7)			6 (1)			158 (2.1)		
		Unknown	142 (6)			86 (6.9)			150 (8.9)			166 (9.8)			69 (11.7)			613 (8.1)		
	**Systemic diseases (yes), n (%)**																			
		Blood disease	56 (2.4)			16 (1.3)			19 (1.1)			19 (1.1)			9 (1.5)			119 (1.6)		
		Brain disease	23 (1)			9 (0.7)			29 (1.7)			11 (0.7)			8 (1.4)			80 (1.1)		
		Collagen disease	10 (0.4)			10 (0.8)			9 (0.5)			8 (0.5)			4 (0.7)			41 (0.5)		
		Heart disease	54 (2.3)			26 (2.1)			27 (1.6)			32 (1.9)			16 (2.7)			155 (2)		
		Kidney disease	48 (2)			31 (2.5)			23 (1.4)			25 (1.5)			9 (1.5)			136 (1.8)		
		Liver disease	45 (1.9)			30 (2.4)			25 (1.5)			24 (1.4)			8 (1.4)			132 (1.7)		
		Malignant tumor	43 (1.8)			16 (1.3)			21 (1.3)			18 (1.1)			4 (0.7)			102 (1.3)		
		Respiratory disease	228 (9.6)			135 (10.9)			160 (9.5)			157 (9.2)			73 (12.4)			753 (9.9)		
		N/A^a^	1931 (81.3)			998 (80.6)			1410 (83.7)			1438 (84.5)			480 (81.5)			6257 (82.4)		
	**Atopic dermatitis (yes), n (%)**			375 (15.8)			207 (16.7)			320 (19)			332 (19.5)			127 (21.6)			1361 (17.9)	
	**Mental illness, n (%)**																			
		No	2165 (91.2)			1098 (88.6)			1474 (87.5)			1461 (85.8)			484 (82.2)			6682 (88)		
		Yes	87 (3.7)			55 (4.4)			101 (6)			121 (7.1)			62 (10.5)			426 (5.6)		
		Previously had	123 (5.2)			86 (6.9)			110 (6.5)			120 (7.1)			43 (7.3)			482 (6.4)		
	**History of dry eye diagnosis, n (%)**																			
		No	1363 (57.4)			616 (49.7)			858 (50.9)			784 (46.1)			250 (42.4)			3871 (51)		
		Yes	499 (21)			305 (24.6)			405 (24)			427 (25.1)			158 (26.8)			1794 (23.6)		
		Unknown	513 (21.6)			318 (25.7)			422 (25)			491 (28.9)			181 (30.7)			1925 (25.4)		

^a^Not applicable.

### JACQLQ Domain II Scores

The JACQLQ Domain II scores are presented in Tables 3 and 4. Mean scores of the total daily activity, total psychological well-being, and total JACQLQ Domain II were 8.9 (SD 9.6), 5.9 (SD 6.7), and 14.8 (SD 15.5) points, respectively.

**Table 3:** JACQLQa Domain II scores stratified by the face scale score category of participants.

JACQLQ Domain II scores		Face scale score 0 (n=667, 8.8%)	Face scale score 1 (n=1864, 24.6%)	Face scale score 2 (n=2755, 36.3%)	Face scale score 3 (n=1849, 24.4%)	Face scale score 4 (n=455, 6%)	Overall (n=7590, 100%)
**Daily activity (0-4), mean (SD)**							
	1. Obstacles to studying, working, and housework	0.2 (0.6)	0.6 (0.9)	1.1 (1.1)	1.8 (1.3)	2.4 (1.5)	1.1 (1.3)
	2. Poor mental concentration	0.2 (0.6)	0.6 (0.9)	1.1 (1.1)	1.9 (1.3)	2.4 (1.4)	1.1 (1.3)
	3. Decreased thinking ability	0.1 (0.5)	0.4 (0.8)	0.9 (1.0)	1.6 (1.2)	2.1 (1.5)	0.9 (1.2)
	4. Impaired reading newspapers and other materials	0.1 (0.5)	0.3 (0.7)	0.6 (0.9)	1.1 (1.2)	1.5 (1.4)	0.6 (1.0)
	5. Poor memory	0.1 (0.5)	0.3 (0.7)	0.5 (0.9)	1.0 (1.2)	1.4 (1.4)	0.6 (1.0)
	6. Limitation of outdoor life such as sports and picnics	0.2 (0.7)	0.4 (0.9)	0.8 (1.2)	1.4 (1.5)	2.0 (1.6)	0.9 (1.3)
	7. Limitation of going out	0.2 (0.6)	0.5 (0.9)	0.9 (1.2)	1.6 (1.5)	2.2 (1.6)	1.0 (1.3)
	8. Obstacles to socializing with people	0.1 (0.5)	0.2 (0.7)	0.6 (1.0)	1.1 (1.3)	1.7 (1.5)	0.6 (1.1)
	9. Interfering with conversations and telephone calls with others	0.1 (0.4)	0.2 (0.6)	0.5 (0.9)	1.0 (1.2)	1.4 (1.5)	0.5 (1.0)
	10. Anxiety about people around you	0.1 (0.3)	0.2 (0.6)	0.5 (0.9)	1.0 (1.3)	1.4 (1.6)	0.5 (1.0)
	11. Sleeping disorder	0.1 (0.4)	0.4 (0.8)	0.8 (1.1)	1.4 (1.4)	2.0 (1.6)	0.9 (1.2)
Total daily activity score (0-44), mean (SD)		1.3 (3.3)	4.1 (5.7)	8.2 (7.7)	14.9 (10.1)	20.4 (12.2)	8.9 (9.6)
**Psychological well-being (0-4), mean (SD)**							
	12. Dullness	0.2 (0.6)	0.4 (0.8)	1.0 (1.2)	1.8 (1.4)	2.5 (1.5)	1.1 (1.3)
	13. Fatigue	0.1 (0.5)	0.5 (0.9)	1.1 (1.2)	1.9 (1.4)	2.6 (1.5)	1.2 (1.3)
	14. Frustrated	0.1 (0.5)	0.3 (0.8)	0.9 (1.1)	1.9 (1.4)	2.6 (1.5)	1.0 (1.3)
	15. Irritable	0.1 (0.5)	0.3 (0.7)	0.8 (1.1)	1.6 (1.4)	2.3 (1.6)	0.9 (1.3)
	16. Depressed	0.0 (0.3)	0.2 (0.7)	0.7 (1.0)	1.6 (1.4)	2.5 (1.5)	0.9 (1.3)
	17. Dissatisfaction with life	0.1 (0.3)	0.2 (0.6)	0.7 (1.0)	1.6 (1.4)	2.4 (1.6)	0.8 (1.2)
Total psychological well-being (0-24), mean (SD)		0.6 (2.1)	2.0 (3.3)	5.3 (5.2)	10.4 (6.9)	14.9 (7.9)	5.9 (6.7)
Total domain II score (0-68), mean (SD)		1.9 (5.0)	6.1 (8.4)	13.4 (11.9)	25.3 (15.9)	35.3 (18.8)	14.8 (15.5)

^a^JACQLQ: Japanese Allergic Conjunctival Disease Quality of Life Questionnaire.

**Table 4:** JACQLQa Domain II scores stratified by stress level scale category of participants.

JACQLQ Domain II scores			Stress level scale 0-2 (n=2375, 31.3%)		Stress level scale 3-4 (n=1239, 16.3%)		Stress level scale 5-6 (n=1685, 22.2%)		Stress level scale 7-8 (n=1702, 22.4%)		Stress level scale 9-10 (n=589, 7.8%)		Overall (n=7590, 100%)
**Daily activity (0-4), mean (SD)**													
	1. Obstacles to studying, working, and housework	0.4 (0.8)		1.0 (0.0)		1.1 (0.0)		1.8 (1.2)		2.6 (1.4)		1.1 (1.3)	
	2. Poor mental concentration	0.5 (0.9)		1.0 (1.0)		1.1 (1.1)		1.8 (1.2)		2.6 (1.3)		1.1 (1.3)	
	3. Decreased thinking ability	0.4 (0.8)		0.8 (1.0)		0.9 (1.1)		1.5 (1.2)		2.2 (1.4)		0.9 (1.2)	
	4. Impaired reading newspapers and other materials	0.3 (0.7)		0.5 (0.9)		0.6 (0.9)		1.0 (1.1)		1.6 (1.4)		0.6 (1.0)	
	5. Poor memory	0.3 (0.7)		0.5 (0.9)		0.6 (1.0)		0.9 (1.1)		1.4 (1.4)		0.6 (1.0)	
	6. Limitation of outdoor life such as sports and picnics	0.4 (0.9)		0.7 (1.1)		0.8 (1.2)		1.4 (1.4)		1.9 (1.6)		0.9 (1.3)	
	7. Limitation of going out	0.4 (1.0)		0.8 (1.2)		0.9 (1.2)		1.6 (1.4)		2.2 (1.6)		1.0 (1.3)	
	8. Obstacles to socializing with people	0.3 (0.7)		0.5 (0.9)		0.6 (1.0)		1.0 (1.2)		1.6 (1.5)		0.6 (1.1)	
	9. Interfering with conversations and telephone calls with others	0.2 (0.6)		0.4 (0.9)		0.5 (0.9)		0.9 (1.1)		1.5 (1.5)		0.5 (1.0)	
	10. Anxiety about people around you	0.2 (0.6)		0.4 (0.9)		0.5 (1.0)		0.9 (1.2)		1.4 (1.5)		0.5 (1.0)	
	11. Sleeping disorder	0.4 (0.9)		0.7 (1.1)		0.8 (1.1)		1.4 (1.4)		2.0 (1.6)		0.9 (1.2)	
Total daily activity score (0-44), mean (SD)			3.6 (5.9)		7.3 (7.3)		8.3 (8.4)		14.1 (9.5)		21.0 (11.2)		8.9 (9.6)
**Psychological well-being (0-4), mean (SD)**													
	12. Dullness	0.4 (0.9)		0.8 (1.1)		1.0 (1.2)		1.7 (1.4)		2.5 (1.5)		1.1 (1.3)	
	13. Fatigue	0.5 (0.9)		1.0 (1.1)		1.1 (1.2)		1.8 (1.4)		2.6 (1.4)		1.2 (1.3)	
	14. Frustrated	0.4 (0.9)		0.8 (1.0)		1.0 (1.2)		1.6 (1.4)		2.6 (1.5)		1.0 (1.3)	
	15. Irritable	0.3 (0.8)		0.7 (1.0)		0.8 (1.2)		1.5 (1.3)		2.4 (1.5)		0.9 (1.3)	
	16. Depressed	0.3 (0.8)		0.6 (1.0)		0.8 (1.2)		1.3 (1.3)		2.4 (1.5)		0.9 (1.3)	
	17. Dissatisfaction with life	0.3 (0.7)		0.6 (1.0)		0.8 (1.1)		1.3 (1.3)		2.2 (1.5)		0.8 (1.2)	
Total psychological well-being (0-24), mean (SD)			2.3 (4.0)		4.4 (5.0)		5.6 (6.0)		9.2 (6.7)		14.8 (7.3)		5.9 (6.7)
Total domain II score (0-68), mean (SD)			5.9 (9.2)		11.7 (11.6)		13.8 (13.5)		23.3 (15.1)		35.9 (17.1)		14.8 (15.5)

^a^JACQLQ: Japanese Allergic Conjunctival Disease Quality of Life Questionnaire.

### MCID Estimates Obtained Using the Anchor-Based Method

Spearman correlation coefficients between severity anchor categories (face scale score and stress level scale score) and the total scores (daily activity, psychological well-being, and total JACQLQ Domain II score) were ≥0.3, and anchors were considered eligible (face scale category: daily activity 0.555, psychological well-being 0.597, and total JACQLQ Domain II score 0.603; stress level scale category: daily activity 0.527, psychological well-being 0.513, and total JACQLQ Domain II score 0.547). MCID estimates calculated using the anchor-based method are shown in Table 5 (range: daily activity 1.0-6.9; psychological well-being 1.2-5.6; and total JACQLQ Domain II score 2.1-12.6).

**Table 5:** Summary of MCIDa estimates.

		Daily activity	Psychological well-being	Total domain II score
**Anchor-based estimates, mean difference**				
	Face scale score 1–0	2.8	1.4	4.2
	Face scale score 2–1	4.1	3.3	7.3
	Face scale score 3–2	6.7	5.1	11.9
	Face scale score 4–3	5.5	4.5	10.0
	Stress level scale 3–4 – 0–2	3.7	2.1	5.8
	Stress level scale 5–6 – 3–4	1.0	1.2	2.1
	Stress level scale 7–8 – 5–6	5.8	3.6	9.5
	Stress level scale 9–10 – 7–8	6.9	5.6	12.6
Anchor-based estimates, median (IQR)		4.8 (3.5-6.0)	3.5 (1.9-4.7)	8.4 (5.4-10.5)
**Distribution-based estimates**				
	0.5 SD	4.8	3.4	7.8
	Standard error of measurement	4.2	3.1	6.4
	MCID range	4.2-6.0	3.1-4.7	6.4-10.5

^a^MCID: minimal clinically important difference.

### MCID Estimates Obtained Using the Distribution-Based Method

Table 5 presents the MCID estimates calculated using the distribution-based method. The MCID estimates based on 0.5 SD of the JACQLQ Domain II scores at baseline were 4.8, 3.4, and 7.8 for daily activity, psychological well-being, and the total JACQLQ Domain II scores, respectively. ICCs were calculated to determine the SEM from the test-retest data of 286 participants (mean age 38.8, SD 14.7 years; 50%, n=143 of the participants were women). ICCs were odds ratio (OR) 0.813 (95% CI 0.769-0.849), OR 0.791 (95% CI 0.743-0.832), and OR 0.841 (95% CI 0.791-0.864) for daily activity, psychological well-being, and the total JACQLQ Domain II scores, respectively (Multimedia Appendix 5). The SEMs calculated using the ICCs were 4.2, 3.1, and 6.4 for daily activity, psychological well-being, and the total JACQLQ Domain II scores, respectively (Table 5).

### Estimation of the MCID Ranges

The SEMs of the JACQLQ Domain II scores were larger than the 25th percentile of the median anchor-based estimates and 0.5 SD, and the SEMs were selected as the lower bound of the MCID ranges. The final suggested MCID ranges for daily activity, psychological well-being, and the total JACQLQ Domain II scores were 4.2-6.0, 3.1-4.7, and 6.4-10.5, respectively (Table 5).

## Discussion

### Principal Results

The implementation of an MCID derived from ePRO data is ideal for assessing subjective symptoms and QoL ascertained by electronic means, despite the similarity to its traditional paper-based counterpart [52]. In this study, we determined the MCID for the app-based JACQLQ Domain II score derived using ePRO data from a previous study that targeted the users of a smartphone app for hay fever research. The MCID reported in this study may improve the evaluation of QoL related to allergic conjunctivitis in patients with hay fever, particularly in remote settings.

### Comparison With Prior Work

This study yielded MCID ranges of 4.2-6.0, 3.1-4.7, and 6.4-10.5 for daily activity, psychological well-being, and the total JACQLQ Domain II scores, respectively. A paper-based JRQLQ study performed in Japan on patients with hay fever who visited a University Hospital [12] previously reported MCID values of 6.0-10.5 for the total QoL score collected from the JRQLQ (mean 8.3; per-item: 0.5). With a total JACQLQ Domain II score-related MCID range of 6.4-10.5 (mean: 8.5; per-item: 0.5), the results of this study are comparable to those of the previous study. Of note, our previous efforts to evaluate and challenge the implementation of the electronic JACQLQ using “AllerSearch” [14] demonstrated that the JACQLQ administered via a smartphone app is sufficiently valid and reliable. This posits a similar potential for the use of app-based and paper-based JACQLQ in clinical practice to assess the QoL affected by hay fever–induced allergic conjunctivitis [14].

In addition to using a large-scale clinical dataset collected through smartphone apps, the MCID was derived from two separate calculations: anchor-based and distribution-based methods [27]. Several internal considerations and recommendations were implemented to improve the accuracy of the MCID calculation. First, the inclusion of PRO data collected from a large pool of participants has been recommended [31], for which our app-based methodology was well-suited. Our study successfully analyzed a large dataset of PRO data provided by 7590 participants. Second, deriving an MCID range by tandem use of both anchor- and distribution-based methods has been recommended for a comprehensive evaluation [27,53], both of which were considered in this study. Furthermore, two distinct anchors, the JACQLQ Domain II face scale and stress level scale, were selected to minimize bias and subjectivity that may stem from a single anchor selected by the research team for the anchor-based calculation. For the distribution-based calculation, both SD and SEM were considered for a multifaceted approach and to better reflect the characteristics of PRO data in the final MCID [4,27]. With the multistep strategy implemented to ensure that the final MCID encapsulates various factors, we believe that the MCID determined in this study is sufficiently accurate for use in a mHealth setting to evaluate subjective symptoms and QoL of hay fever and co-occurring allergic conjunctivitis. Using the MCIDs suggested in this study, providers may be able to accurately assess and compare patients’ dynamic ePROs on hay fever-related symptoms and QoL submitted through commonplace smart devices, which should further enable improved evaluation of treatment responses without necessitating frequent visits to medical facilities.

In this study, we aimed to establish new MCID values for app-based JACQLQ Domain II scores by analyzing a large dataset. The originality of this research lies in the use of crowdsourced data collected through a smartphone-specific QoL assessment tool tailored for allergic conjunctivitis [3,4,37]. This approach allowed us to determine the MCID values suitable for app-based QoL assessments, which could not be achieved using traditional paper-based questionnaires. In addition, by using both anchor-based and distribution-based methods, we were able to derive more robust MCID ranges [27,53]. The MCID ranges for daily activity (4.2-6.0), psychological well-being (3.1-4.7), and total JACQLQ Domain II scores (6.4-10.5) closely matched previously reported values from paper-based assessments, demonstrating their clinical relevance [12]. The newly established MCID values for the app-based JACQLQ Domain II score offer a novel approach for assessing the treatment response in patients with allergic conjunctivitis using smartphone apps, potentially allowing for continuous monitoring and assessment of symptoms without the need for frequent in-person visits. The use of app-based interventions also holds promise for apps in telemedicine [54]. The transition from traditional clinical assessments to remote app-based assessments could increase patient accessibility, facilitate early detection and intervention, help prevent disease progression, potentially reduce health care costs, and improve the overall efficiency of allergic conjunctivitis management [55]. In addition, compared to traditional paper-based methods, using smartphones allows data to be stored within the app or in cloud environments, enabling patients to monitor the progression of their symptoms. Providing patients with feedback on their treatment progress can encourage behavioral changes, leading to improved adherence to hay fever prevention measures and medication [56]. This approach may result in more favorable treatment outcomes than traditional face-to-face consultations [57]. Allergic conjunctivitis caused by hay fever is influenced by environmental factors in the patient’s daily living environment and lifestyle habits. By providing feedback to physicians based on real-time monitoring information obtained from patients through a smartphone app, physicians may be able to offer tailored lifestyle counseling and treatment interventions appropriate for each patient. The integration of smartphone-based assessments and established MCID values provides a more efficient and accessible approach for managing allergic conjunctivitis, potentially improving patient outcomes and streamlining clinical care.

### Limitations

This study has some limitations. First, the data of interest in this study were ePROs, and every variable analyzed to assess hay fever status was based solely on subjective ePRO data. Objective data, such as assessments through physical examination and clinical test results, were not considered. It is unclear from the current results whether a correlation between changes in QoL scores above the MCID and objective data exists. Future studies could further evaluate the validity of the MCID determined in this study by comparing it with objective clinical findings, such as the degree of conjunctival congestion, edema, and follicle presence. Second, the data used to determine the MCID were obtained from “AllerSearch” users in Japan. We recommend careful consideration of the user interface and layout of any non-“AllerSearch” app-based JACQLQ, as well as a target population with a high proportion of non-Japanese individuals when applying the reported MCIDs. Third, a significant portion of our ePRO data points, such as those on subjective symptoms and QoL related to hay fever, were collected between February and March. Although the dataset includes ePROs on hay fever data collected throughout the year, it is possible that the MCID reported in this study is weighted toward cedar and cypress pollen–related hay fever symptoms and QoL changes, which represent a major proportion of the Japanese hay fever population. Fourth, the anchor specified in this study was selected based on cross-sectional study data. Therefore, the anchor may not necessarily correlate with longitudinal changes in hay fever symptoms. In addition, the MCID calculated in this study may not be strictly equivalent to the minimally important change. Therefore, it is necessary to evaluate the validity of the MCID calculated in this study in future longitudinal studies. Fifth, this study determined the SEM for the distribution-based method using the ICC. The ICC was derived from comparisons between baseline and next-day measurements to assess the test-retest reliability of the questionnaire while avoiding changes in symptoms caused by external factors such as continued exposure to pollen, changes in medication use, and variations in pollen levels. Our test-retest analysis involves a short interval between measurements, which could lead to the possibility of recall bias. However, because the study participants entered the data voluntarily, they may have reported based on their symptoms at the time rather than their previous answers, potentially reducing the risk of recall bias.

### Conclusions

We report MCID ranges of 4.2-6.0, 3.1-4.7, and 6.4-10.5 for daily activity, psychological well-being, and the total JACQLQ Domain II scores, respectively, that have been derived from a large-scale ePRO dataset collected through an in-house smartphone app, “AllerSearch,” and finally calculated using a multifaceted approach. The MCIDs determined in this study potentially have clinical application in the establishment of standard references to enable the remote assessment and monitoring of hay fever and co-occurring allergic conjunctivitis and the implementation of the principles of mHealth in clinical practice. In addition, combining an MCID derived from an entirely ePRO-based methodology with an app-administered JACQLQ could facilitate fully remote monitoring of symptoms and QoL changes in patients with hay fever–related allergic conjunctivitis, and may enable better assessment of day-to-day treatment effects for an extended and improved disease monitoring.

## Appendix

Multimedia Appendix 1 Survey questions.

Multimedia Appendix 2 Daily hay-fever subjective symptom questionnaire.

Multimedia Appendix 3 JACQLQ (Japanese Allergic Conjunctival Disease QoL Questionnaire) Domains II and III.

Multimedia Appendix 4 Flowchart of participant enrollment. JACQLQ: Japanese Allergic Conjunctival Disease Quality of Life Questionnaire.

Multimedia Appendix 5 Test–retest reliability of QoL scores.

## Abbreviations

ePRO: electronic patient-reported outcome
ICC: intraclass correlation coefficient
JACQLQ: Japanese Allergic Conjunctival Disease QoL Questionnaire
JRQLQ: Japanese Rhinoconjunctivitis Quality of Life Questionnaire
MCID: minimal clinically important difference
mHealth: mobile health
OR: odds ratio
PRO: patient-reported outcome
QoL: quality of life
SEM: standard error of measurement

## Data Availability

The datasets generated during or analyzed during this study are available from the corresponding author upon reasonable request.

## References

[1] Sakashita M, Hirota T, Harada M, Nakamichi R, Tsunoda T, Osawa Y, Kojima A, Okamoto M, Suzuki D, Kubo S, Imoto Y, Nakamura Y, Tamari M, Fujieda S (2010). Prevalence of allergic rhinitis and sensitization to common aeroallergens in a Japanese population. Int Arch Allergy Immunol.

[2] Mortimer K, Lesosky M, García-Marcos L, Asher MI, Pearce N, Ellwood E, Bissell K, El Sony A, Ellwood P, Marks GB, Martínez-Torres A, Morales E, Perez-Fernandez V, Robertson S, Rutter CE, Silverwood RJ, Strachan DP, Chiang C (2022). The burden of asthma, hay fever and eczema in adults in 17 countries: GAN phase I study. Eur Respir J.

[3] Inomata T, Nakamura M, Iwagami M, Sung J, Nakamura M, Ebihara N, Fujisawa K, Muto K, Nojiri S, Ide T, Okano M, Okumura Y, Fujio K, Fujimoto K, Nagao M, Hirosawa K, Akasaki Y, Murakami A (2021). Symptom-based stratification for hay fever: a crowdsourced study using the smartphone application allerSearch. Allergy.

[4] Inomata T, Nakamura M, Iwagami M, Sung J, Nakamura M, Ebihara N, Fujisawa K, Muto K, Nojiri S, Ide T, Okano M, Okumura Y, Fujio K, Fujimoto K, Nagao M, Hirosawa K, Akasaki Y, Murakami A (2022). Individual characteristics and associated factors of hay fever: a large-scale mHealth study using AllerSearch. Allergol Int.

[5] Mimura T, Ichinose T, Yamagami S, Fujishima H, Kamei Y, Goto M, Takada S, Matsubara M (2014). Airborne particulate matter (PM2.5) and the prevalence of allergic conjunctivitis in Japan. Sci Total Environ.

[6] Lee YJ, Han SJ, Lee H, Kim JS, Seo KY (2016). Development of allergic conjunctivitis induced by house dust mite extract from dermatophagoides pteronyssinus. Invest Ophthalmol Vis Sci.

[7] Bowatte G, Lodge C, Lowe AJ, Erbas B, Perret J, Abramson MJ, Matheson M, Dharmage SC (2015). The influence of childhood traffic-related air pollution exposure on asthma, allergy and sensitization: a systematic review and a meta-analysis of birth cohort studies. Allergy.

[8] Inomata T, Sung J, Fujio K, Nakamura M, Akasaki Y, Nagino K, Okumura Y, Iwagami M, Fujimoto K, Ebihara N, Nakamura M, Midorikawa-Inomata A, Shokirova H, Huang T, Hirosawa K, Miura M, Ohno M, Morooka Y, Iwata N, Iwasaki Y, Murakami A (2023). Individual multidisciplinary clinical phenotypes of nasal and ocular symptoms in hay fever: crowdsourced cross-sectional study using AllerSearch. Allergol Int.

[9] Miyazaki D, Fukagawa K, Okamoto S, Fukushima A, Uchio E, Ebihara N, Shoji J, Namba K, Shimizu Y (2020). Epidemiological aspects of allergic conjunctivitis. Allergol Int.

[10] Kimchi N, Bielory L (2020). The allergic eye: recommendations about pharmacotherapy and recent therapeutic agents. Curr Opin Allergy Clin Immunol.

[11] Platt M (2014). Pharmacotherapy for allergic rhinitis. Int Forum Allergy Rhinol.

[12] Higaki T, Okano M, Kariya S, Fujiwara T, Haruna T, Hirai H, Murai A, Gotoh M, Okubo K, Yonekura S, Okamoto Y, Nishizaki K (2013). Determining minimal clinically important differences in Japanese cedar/cypress pollinosis patients. Allergol Int.

[13] Miyazaki D, Fukushima A, Uchio E, Shoji J, Namba K, Ebihara N, Takamura E, Fukuda K, Matsuda A, Okamoto S, Fukagawa K, Fujishima H, Ohno S, Ohashi Y (2022). Executive summary: Japanese guidelines for allergic conjunctival diseases 2021. Allergol Int.

[14] Akasaki Y, Inomata T, Sung J, Okumura Y, Fujio K, Miura M, Hirosawa K, Iwagami M, Nakamura M, Ebihara N, Nakamura M, Ide T, Nagino K, Murakami A (2022). Reliability and validity of electronic patient-reported outcomes using the smartphone app allersearch for hay fever: prospective observational study. JMIR Form Res.

[15] Devillier P, Chassany O, Vicaut E, de Beaumont O, Robin B, Dreyfus J, Bousquet P (2014). The minimally important difference in the rhinoconjunctivitis total symptom score in grass-pollen-induced allergic rhinoconjunctivitis. Allergy.

[16] U.S. Department of HealthHuman Services FDA Center for Drug EvaluationResearch, U.S. Department of HealthHuman Services FDA Center for Biologics EvaluationResearch, U.S. Department of HealthHuman Services FDA Center for DevicesRadiological Health (2006). Guidance for industry: patient-reported outcome measures: use in medical product development to support labeling claims: draft guidance. Health Qual Life Outcomes.

[17] Okumura Y, Inomata T, Iwata N, Sung J, Fujimoto K, Fujio K, Midorikawa-Inomata A, Miura M, Akasaki Y, Murakami A (2020). A review of dry eye questionnaires: measuring patient-reported outcomes and health-related quality of life. Diagnostics (Basel).

[18] Bottomley A, Jones D, Claassens L (2009). Patient-reported outcomes: assessment and current perspectives of the guidelines of the food and drug administration and the reflection paper of the European medicines agency. Eur J Cancer.

[19] Inomata T, Iwagami M, Nakamura M, Shiang T, Fujimoto K, Okumura Y, Iwata N, Fujio K, Hiratsuka Y, Hori S, Tsubota K, Dana R, Murakami A (2020). Association between dry eye and depressive symptoms: large-scale crowdsourced research using the DryEyeRhythm iPhone application. Ocul Surf.

[20] Inomata T, Nakamura M, Iwagami M, Midorikawa-Inomata A, Sung J, Fujimoto K, Okumura Y, Eguchi A, Iwata N, Miura M, Fujio K, Nagino K, Hori S, Tsubota K, Dana R, Murakami A (2020). Stratification of individual symptoms of contact lens-associated dry eye using the iPhone app DryEyeRhythm: crowdsourced cross-sectional study. J Med Internet Res.

[21] Nagino K, Okumura Y, Akasaki Y, Fujio K, Huang T, Sung J, Midorikawa-Inomata A, Fujimoto K, Eguchi A, Hurramhon S, Yee A, Miura M, Ohno M, Hirosawa K, Morooka Y, Murakami A, Kobayashi H, Inomata T (2023). Smartphone app-based and paper-based patient-reported outcomes using a disease-specific questionnaire for dry eye disease: randomized crossover equivalence study. J Med Internet Res.

[22] Tsiakiri A, Koutzmpi V, Megagianni S, Toumaian M, Geronikola N, Despoti A, Kanellopoulou S, Arampatzi X, Margioti E, Davila A, Zoi P, Kalligerou F, Liozidou A, Tsapanou A, Sakka P (2024). Remote neuropsychological evaluation of older adults. Appl Neuropsychol Adult.

[23] Bonini N, Vitolo M, Imberti JF, Proietti M, Romiti GF, Boriani G, Johnsen SP, Guo Y, Lip GYH (2022). Mobile health technology in atrial fibrillation. Expert Rev Med Devices.

[24] Eguchi A, Inomata T, Nakamura M, Nagino K, Iwagami M, Sung J, Midorikawa-Inomata A, Okumura Y, Fujio K, Fujimoto K, Miura M, Akasaki Y, Shokirova H, Hirosawa K, Kuwahara M, Zhu J, Dana R, Murakami A, Kobayashi H (2021). Heterogeneity of eye drop use among symptomatic dry eye individuals in Japan: large-scale crowdsourced research using DryEyeRhythm application. Jpn J Ophthalmol.

[25] Inomata T, Nakamura M, Sung J, Midorikawa-Inomata A, Iwagami M, Fujio K, Akasaki Y, Okumura Y, Fujimoto K, Eguchi A, Miura M, Nagino K, Shokirova H, Zhu J, Kuwahara M, Hirosawa K, Dana R, Murakami A (2021). Smartphone-based digital phenotyping for dry eye toward P4 medicine: a crowdsourced cross-sectional study. NPJ Digital Med.

[26] Nagino K, Okumura Y, Yamaguchi M, Sung J, Nagao M, Fujio K, Akasaki Y, Huang T, Hirosawa K, Iwagami M, Midorikawa-Inomata A, Fujimoto K, Eguchi A, Okajima Y, Kakisu K, Tei Y, Yamaguchi T, Tomida D, Fukui M, Yagi-Yaguchi Y, Hori Y, Shimazaki J, Nojiri S, Morooka Y, Yee A, Miura M, Ohno M, Inomata T (2023). Diagnostic ability of a smartphone app for dry eye disease: protocol for a multicenter, open-label, prospective, and cross-sectional study. JMIR Res Protoc.

[27] Sedaghat AR (2019). Understanding the minimal clinically important difference (MCID) of patient-reported outcome measures. Otolaryngol Head Neck Surg.

[28] Bhardwaj SS, Camacho F, Derrow A, Fleischer AB, Feldman SR (2004). Statistical significance and clinical relevance: the importance of power in clinical trials in dermatology. Arch Dermatol.

[29] Kerezoudis P, Yost KJ, Tombers NM, Celda MP, Carlson ML, Link MJ (2019). Defining the minimal clinically important difference for patients with vestibular schwannoma: are all quality-of-life scores significant?. Neurosurgery.

[30] Jaeschke R, Singer J, Guyatt GH (1989). Measurement of health status. ascertaining the minimal clinically important difference. Control Clin Trials.

[31] Wang YC, Hart DL, Stratford PW, Mioduski JE (2011). Baseline dependency of minimal clinically important improvement. Phys Ther.

[32] Kirtsreesakul V, Somjareonwattana P, Ruttanaphol S (2009). The correlation between nasal symptom and mucociliary clearance in allergic rhinitis. Laryngoscope.

[33] Juniper EF, Thompson AK, Ferrie PJ, Roberts JN (1999). Validation of the standardized version of the rhinoconjunctivitis quality of life questionnaire. J Allergy Clin Immunol.

[34] Fukagawa K (2014). How to use Japanese allergic conjunctival disease quality-of-life questionnaire (JACQLQ). Arerugi.

[35] Fukagawa K (2017). JACQLQ (Japanese allergic conjunctival diseases quality of life questionnaire). Arerugi.

[36] Nagino K, Sung J, Midorikawa-Inomata A, Eguchi A, Fujimoto K, Okumura Y, Yee A, Fujio K, Akasaki Y, Huang T, Miura M, Hurramhon S, Hirosawa K, Ohno M, Morooka Y, Kobayashi H, Inomata T (2023). The minimal clinically important difference of app-based electronic patient-reported outcomes for hay fever. Clin Transl Allergy.

[37] Fujio K, Inomata T, Fujisawa K, Sung J, Nakamura M, Iwagami M, Muto K, Ebihara N, Nakamura M, Okano M, Akasaki Y, Okumura Y, Ide T, Nojiri S, Nagao M, Fujimoto K, Hirosawa K, Murakami A (2022). Patient and public involvement in mobile health-based research for hay fever: a qualitative study of patient and public involvement implementation process. Res Involve Engage.

[38] (2020). AllerSearch online media.

[39] AllerSearch account on X (formerly Twitter) website.

[40] AllerSearch account on Facebook website.

[41] Inomata T, Sung J, Nakamura M, Fujisawa K, Muto K, Ebihara N, Iwagami M, Nakamura M, Fujio K, Okumura Y, Okano M, Murakami A (2020). New medical big data for P4 medicine on allergic conjunctivitis. Allergol Int.

[42] Inomata T, Sung J, Nakamura M, Iwagami M, Akasaki Y, Fujio K, Nakamura M, Ebihara N, Ide T, Nagao M, Okumura Y, Nagino K, Fujimoto K, Eguchi A, Hirosawa K, Midorikawa-Inomata A, Muto K, Fujisawa K, Kikuchi Y, Nojiri S, Murakami A (2023). Using the AllerSearch smartphone app to assess the association between dry eye and hay fever: mHealth-based cross-sectional study. J Med Internet Res.

[43] Fukagawa K, Fujishima H, Fukushima A, Sumi T, Okamoto S, Shoji J, Satake Y, Ohno S, Namba K, Kitaichi N, Ebihara N, Takahashi H, Kumagai N, Uchino Y, Uchino M, Murayama K, Sakata M, Uchio E, Takamura E, Ohashi Y, Ohkubo K, Satoh T (2012). A quality of life questionnaire for Japanese allergic conjunctival disease. Nippon Ganka Gakkai Zasshi.

[44] Ousmen A, Touraine C, Deliu N, Cottone F, Bonnetain F, Efficace F, Brédart A, Mollevi C, Anota A (2018). Distribution- and anchor-based methods to determine the minimally important difference on patient-reported outcome questionnaires in oncology: a structured review. Health Qual Life Outcomes.

[45] Revicki D, Hays RD, Cella D, Sloan J (2008). Recommended methods for determining responsiveness and minimally important differences for patient-reported outcomes. J Clin Epidemiol.

[46] Farrar JT, Young JP, LaMoreaux L, Werth JL, Poole RM (2001). Clinical importance of changes in chronic pain intensity measured on an 11-point numerical pain rating scale. Pain.

[47] Carlson ML, Tveiten ØV, Yost KJ, Lohse CM, Lund-Johansen M, Link MJ (2015). The minimal clinically important difference in vestibular schwannoma quality-of-life assessment. Otolaryngol Head Neck Surg.

[48] Stark RG, Reitmeir P, Leidl R, König HH (2010). Validity, reliability, and responsiveness of the EQ-5D in inflammatory bowel disease in Germany. Inflamm Bowel Dis.

[49] Norman GR, Sloan JA, Wyrwich KW (2003). Interpretation of changes in health-related quality of life: the remarkable universality of half a standard deviation. Med Care.

[50] Asher AL, Kerezoudis P, Mummaneni PV, Bisson EF, Glassman SD, Foley KT, Slotkin JR, Potts EA, Shaffrey ME, Shaffrey CI, Coric D, Knightly JJ, Park P, Fu K, Devin CJ, Archer KR, Chotai S, Chan AK, Virk MS, Bydon M (2018). Defining the minimum clinically important difference for grade I degenerative lumbar spondylolisthesis: insights from the quality outcomes database. Neurosurg Focus.

[51] Sagberg LM, Jakola AS, Solheim O (2014). Quality of life assessed with EQ-5D in patients undergoing glioma surgery: what is the responsiveness and minimal clinically important difference?. Qual Life Res.

[52] Coons SJ, Gwaltney CJ, Hays RD, Lundy JJ, Sloan JA, Revicki DA, Lenderking WR, Cella D, Basch E (2009). Recommendations on evidence needed to support measurement equivalence between electronic and paper-based patient-reported outcome (PRO) measures: ISPOR ePRO good research practices task force report. Value Health.

[53] Barnes ML, Vaidyanathan S, Williamson PA, Lipworth BJ (2010). The minimal clinically important difference in allergic rhinitis. Clin Exp Allergy.

[54] Nagino K, Sung J, Midorikawa-Inomata A, Eguchi A, Fujimoto K, Okumura Y, Miura M, Yee A, Hurramhon S, Fujio K, Akasaki Y, Hirosawa K, Huang T, Ohno M, Morooka Y, Zou X, Kobayashi H, Inomata T (2023). Clinical utility of smartphone applications in ophthalmology: a systematic review. Ophthalmol Sci.

[55] Snoswell CL, North JB, Caffery LJ (2020). Economic advantages of telehealth and virtual health practitioners: return on investment analysis. JMIR Perioper Med.

[56] Eaton CK, McWilliams E, Yablon D, Kesim I, Ge R, Mirus K, Sconiers T, Donkoh A, Lawrence M, George C, Morrison ML, Muther E, Oates GR, Sathe M, Sawicki GS, Snell C, Riekert K (2024). Cross-cutting mHealth behavior change techniques to support treatment adherence and self-management of complex medical conditions: systematic review. JMIR Mhealth Uhealth.

[57] Etminani K, Engström AT, Göransson C, Sant'Anna A, Nowaczyk S (2020). How behavior change strategies are used to design digital interventions to improve medication adherence and blood pressure among patients with hypertension: systematic review. J Med Internet Res.

