# Supplementary material for: Minimal Clinically Important Differences With the Outcomes of the App-Based Japanese Allergic Conjunctival Diseases Quality of Life Questionnaire: Cross-Sectional Observational Study
Source: JMIR Form Res. 2024 Nov 26;8:e60731. doi: 10.2196/60731 (PMC11632287; doi:10.2196/60731)
Supplement: Multimedia Appendix 1 [file formative_v8i1e60731_app1.docx]

| Questions | Variables | Details of variables |
| --- | --- | --- |
|  |  |  |
| **User characteristics** |  |  |
|  | Age | Integer input, years |
|  | Sex | Choose one {“Man,” “Woman”} |
|  | Height | Integer input, cm |
|  | Weight | Integer input, kg |
| **Medical history** |  |  |
| **Have you ever been diagnosed with hypertension?** | Medicated hypertension | Choose one {“No,” “I am being treated for hypertension,” “I have untreated hypertension,” “I do not know”} |
| **Have you ever been diagnosed with diabetes?** | Diabetes (HbA1c level) | Choose one {“Yes,” “No,” “I do not know”}. If “Yes,” scale bar input of HbA1c level (5–15%) |
| **Have you experienced any of the following illnesses?** | Systemic diseases | Multiple choice among {“Heart disease,” “Respiratory disease,” “Brain disease,” “Liver disease,” “Kidney disease,” 'Blood disease,” “Malignant tumor,” “Collagen disease,” “N/A”} |
| **If you have eczema (atopic dermatitis), when did it start?** | Atopic dermatitis | Choose one {“Infancy (before 1 year of age),” “Early childhood (age 1–6 years),” “Middle childhood (age 7–12 years),” Early adolescence (after the age of 13 years)”} |
| **Do you have any mental illness?** | Mental illness | Choose one {“No,” “Yes,” “Previously had”} |
| **Do you have any of the following mental illnesses?** | Mental illness | If “Yes” in the previous question, multiple choice among {“Depression,” “Schizophrenia,” “Other mental illness”} |
| **Have you ever been diagnosed with dry eye disease?** | Dry eye disease | Choose one {“No,” “Yes,” “I do not know”} |
| **Hay fever** |  |  |
| **Do you have hay fever?** | Hay fever | Choose one {“Yes,” “No,” “Unknown”} |
| **Please tell us which of the following hay fever prevention methods you have been using.** | Preventive behavior | Multiple choice among {“Mask,” “Eye drops,” “Nasal spray/drops,” “Medication,” “Air purifier,” “Glasses and goggles,” “Other,” “Not using any”} |

N/A, not applicable.

Japanese Allergic Conjunctival Disease Quality of Life Questionnaire.
