# Supplementary material for: Minimal Clinically Important Differences With the Outcomes of the App-Based Japanese Allergic Conjunctival Diseases Quality of Life Questionnaire: Cross-Sectional Observational Study
Source: JMIR Form Res. 2024 Nov 26;8:e60731. doi: 10.2196/60731 (PMC11632287; doi:10.2196/60731)
Supplement: Multimedia Appendix 2 [file formative_v8i1e60731_app2.docx]

| Questions | Variables | Details of variables |
| --- | --- | --- |
|  |  |  |
| **Nasal symptom score** |  |  |
| **Please rate how your rhinorrhea has been over the past 24 hours?** | NSS item 1 | Choose one {“No symptoms,” “Mild symptoms (symptoms clearly present but easily tolerated,” “Moderate symptoms (symptoms bothersome but tolerable),” “Severe symptoms (symptoms difficult to tolerate—interfere with activities)”} |
| **Please rate how your nasal congestion has been over the past 24 hours?** | NSS item 2 |  |
| **Please rate how your nasal itching has been over the past 24 hours?** | NSS item 3 |  |
| **Please rate how your sneezing has been over the past 24 hours?** | NSS item 4 |  |
| **How severely does hay fever affect your daily activities?** | NSS item 5 |  |
| **How much eye itching did you experience over the past 24 hours?** | NNSS item 1 |  |
| **How much eye-watering did you experience over the past 24 hours?** | NNSS item 2 |  |
| **How much eye redness did you experience over the past 24 hours?** | NNSS item 3 |  |
| **How much itching of the ear and nose did you experience over the past 24 hours?** | NNSS item 4 |  |
| **Please tell your stress level on a scale of one to five** | Stress level scale | Slider input, 0–10 |

NSS, nasal symptom score; NNSS, non-nasal symptom score.
