# Supplementary material for: Minimal Clinically Important Differences With the Outcomes of the App-Based Japanese Allergic Conjunctival Diseases Quality of Life Questionnaire: Cross-Sectional Observational Study
Source: JMIR Form Res. 2024 Nov 26;8:e60731. doi: 10.2196/60731 (PMC11632287; doi:10.2196/60731)
Supplement: Multimedia Appendix 3 [file formative_v8i1e60731_app3.docx]

| Questions | Variables | Details of variables |
| --- | --- | --- |
|  | |  |
| **JACQLQ Domain II** | |  |
| **Please check the degree to which the following quality of life questions was most severe in the last 1–2 weeks owing to nasal/eye symptoms. Please check “none” for items that are clearly not related to nasal/ocular symptoms.** | |  |
| **Obstacles to studying, working, and housework** | QoL item 1 | Choose one {“None, “Mild,” “Soft,” “Somewhat severe” “Severe,” “Very severe”} |
| **Poor mental concentration** | QoL item 2 |  |
| **Decreased thinking ability** | QoL item 3 |  |
| **Impaired reading newspapers**  **and other materials** | QoL item 4 |  |
| **Poor memory** | QoL item 5 |  |
| **Limitation of outdoor life such as sports and picnics** | QoL item 6 |  |
| **Limitation of going out** | QoL item 7 |  |
| **Obstacles to socializing with people** | QoL item 8 |  |
| **Interfering with conversations and telephone calls with others** | QoL item 9 |  |
| **Anxiety about people around you** | QoL item 10 |  |
| **Sleeping disorder** | QoL item 11 |  |
| **Dullness** | QoL item 12 |  |
| **Fatigue** | QoL item 13 |  |
| **Frustrated** | QoL item 14 |  |
| **Irritable** | QoL item 15 |  |
| **Depressed** | QoL item 16 |  |
| **Dissatisfaction with life** | QoL item 17 |  |
| **JACQLQ Domain III** | |  |
| **Please check the number on the face that describes your general condition (including symptoms, life, and feelings) in the last 1–2 weeks.** | QoL item 18 (face scale score) | Choose one from face scale |

QoL, quality of life; JACQLQ, Japanese Allergic Conjunctival Disease Quality of Life Questionnaire.
