# Supplementary material for: Minimal Clinically Important Differences With the Outcomes of the App-Based Japanese Allergic Conjunctival Diseases Quality of Life Questionnaire: Cross-Sectional Observational Study
Source: JMIR Form Res. 2024 Nov 26;8:e60731. doi: 10.2196/60731 (PMC11632287; doi:10.2196/60731)
Supplement: Multimedia Appendix 5 [file formative_v8i1e60731_app5.docx]

| Questionnaires | Number of items | Day 1 | Day 2 | ICC (95% CI) |
| --- | --- | --- | --- | --- |
|  |  | Mean (SD) | Mean (SD) |  |
|  |  |  |  |  |
| **Total daily activity score, 0–44** | 11 | 6.7 (9.3) | 6.0 (9.1) | 0.813 (0.769–0.849) |
| **Total psychological well-being, 0–24** | 6 | 4.6 (6.4) | 4.0 (6.2) | 0.791 (0.743–0.832) |
| **Total domain II score, 0–68** | 17 | 11.3 (15.0) | 10.0 (14.9) | 0.831 (0.791–0.864) |

CI, confidence interval; ICC, intraclass correlation coefficient; QoL, quality of life; SD, standard deviation.
